# Targeting USP8 Inhibits O‐GlcNAcylation of SLC7A11 to Promote Ferroptosis of Hepatocellular Carcinoma via Stabilization of OGT

**DOI:** 10.1002/advs.202302953

**Published:** 2023-10-22

**Authors:** Jianing Tang, Guo Long, Kuan Hu, Desheng Xiao, Shuang Liu, Liang Xiao, Ledu Zhou, Yongguang Tao

**Affiliations:** ^1^ Department of Liver Surgery Xiangya Hospital Central South University 110 Xiangya Road Changsha Hunan 410078 China; ^2^ National Clinical Research Center for Geriatric Disorders Xiangya Hospital Central South University Changsha Hunan 410008 China; ^3^ Department of Pathology Xiangya Hospital Central South University Changsha Hunan 410078 China; ^4^ Department of Oncology Institute of Medical Sciences National Clinical Research Center for Geriatric Disorders Xiangya Hospital Central South University Changsha Hunan 410078 China; ^5^ Department of Pathology Key Laboratory of Carcinogenesis and Cancer Invasion (Ministry of Education) Xiangya Hospital Central South University 110 Xiangya Road Changsha Hunan 410078 China; ^6^ NHC Key Laboratory of Carcinogenesis (Central South University) Cancer Research Institute and School of Basic Medicine Central South University 110 Xiangya Road Changsha Hunan 410078 China; ^7^ Department of Thoracic Surgery Hunan Key Laboratory of Early Diagnosis and Precision Therapy in Lung Cancer and Hunan Key Laboratory of Tumor Models and Individualized Medicine Second Xiangya Hospital Central South University 110 Xiangya Road Changsha Hunan 410011 China; ^8^ Hunan Key Laboratory of Cancer Metabolism Hunan Cancer Hospital and Affiliated Cancer Hospital of Xiangya School of Medicine Central South University 110 Xiangya Road Changsha Hunan 410078 China

**Keywords:** deubiquitylation, ferroptosis, O‐GlcNAcylation, OGT, USP8

## Abstract

Hepatocellular carcinoma (HCC) is a lethal and aggressive human malignancy. The present study examins the anti‐tumor effects of deubiquitylating enzymes (DUB) inhibitors in HCC. It is found that the inhibitor of ubiquitin specific peptidase 8 (USP8) and DUB‐IN‐3 shows the most effective anti‐cancer responses. Targeting USP8 inhibits the proliferation of HCC and induces cell ferroptosis. In vivo xenograft and metastasis experiments indicate that inhibition of USP8 suppresses tumor growth and lung metastasis. DUB‐IN‐3 treatment or USP8 depletion decrease intracellular cystine levels and glutathione biosynthesis while increasing the accumulation of reactive oxygen species (ROS). Mechanistical studies reveal that USP8 stabilizes O‐GlcNAc transferase (OGT) via inhibiting K48‐specific poly‐ubiquitination process on OGT protein at K117 site, and STE20‐like kinase (SLK)‐mediated S716 phosphorylation of USP8 is required for the interaction with OGT. Most importantly, OGT O‐GlcNAcylates solute carrier family 7, member 11 (SLC7A11) at Ser26 in HCC cells, which is essential for SLC7A11 to import the cystine from the extracellular environment. Collectively, this study demonstrates that pharmacological inhibition or knockout of USP8 can inhibit the progression of HCC and induce ferroptosis via decreasing the stability of OGT, which imposes a great challenge that targeting of USP8 is a potential approach for HCC treatment.

## Graphical abstract

Targeting USP8 suppresses HCC progression and induces ferroptosis. USP8 stabilizes OGT to O‐GlcNAcylate SLC7A11, which is essential for SLC7A11 to import the cystine from the extracellular environment. Targeting USP8 may be a promising approach for the treatment of HCC.

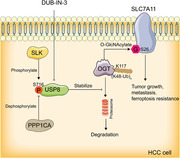

## 1 Introduction

Hepatocellular carcinoma (HCC) is the most prevalent subtype of primary hepatic carcinoma, accounting for approximately 90% of all primary liver cancers.^[^ ^1^ ^]^ HCC is an aggressive malignant tumor and is usually undetectable. It is known for its high malignance, rapid progression, high metastatic potency, easy recurrence, and poor clinical outcomes. The five‐year survival rate of HCC patients is less than 15% even after surgical resection or comprehensive treatment. Recent advances in major clinical interventions, including surgery, transplantation, chemotherapy, radiotherapy, drug‐targeted therapy, and immunotherapy have improved the prognosis of HCC.^[^ ^2^, ^3^, ^4^ ^]^ However, it is still a therapeutic challenge to treat patients with advanced stages of HCC. Therefore, it is an urgent need to develop effective approaches to treat patients with HCC.

Ubiquitination is a post‐translational modification that is essential for cellular homeostatic maintenance.^[^ ^5^ ^]^ Accumulating evidence has indicated that ubiquitination is involved in processes such as cell‐cycle progression, cell survival, apoptosis, DNA repair, and antigen presentation.^[^ ^6^, ^7^, ^8^ ^]^ The major part of ubiquitination is mediated by three enzymes: a ubiquitin‐activating enzyme (E1), a ubiquitin‐conjugating enzyme (E2), and a ubiquitin ligase (E3).^[^ ^9^ ^]^ The dysregulation of ubiquitination is associated with the occurrence and progression of human cancers.^[^ ^10^, ^11^ ^]^ It should be noted that the ubiquitination of cellular proteins is a reversible and dynamic process, which is precisely and orchestrated determined by several E3 ubiquitin ligases and deubiquitylating enzymes (DUBs).^[^ ^12^, ^13^, ^14^ ^]^ E3 ubiquitin ligases recognize specific substrates and directly catalyze or promote ubiquitin transfer to substrate lysine residues.^[^ ^10^ ^]^ While DUBs can negatively regulate this process by cleaving ubiquitin from substrate proteins.^[^ ^15^ ^]^

It has been hypothesized that approximately 100 DUBs are encoded by the human genome.^[^ ^16^, ^17^ ^]^ Based on sequence and catalytic domain, DUBs are classified into six families: ubiquitin‐specific proteases (USPs), ubiquitin COOH‐terminal hydrolases (UCH), Machado–Joseph disease proteases (MJDs), motif interacting with ubiquitin‐containing novel DUB family (MINDY), ovarian tumor proteases (OTUs), and Jab1/Mov34/Mpr1 (JAMM) metalloproteases.^[^ ^18^, ^19^ ^]^ Accumulating studies implicate DUBs in tumorigenesis at multiple levels. Some DUBs, including ubiquitin carboxyl‐terminal esterase L1 (UCHL1), BRCA1 associated protein‐1 (BAP1), and cylindromatosis (CYLD), are described as displaying intrinsic oncogenic or tumor suppressor activities.^[^ ^20^ ^]^ A number of DUBs, such as ubiquitin specific peptidase 1 (USP1), ubiquitin specific peptidase 7 (USP7), and ubiquitin carboxyl‐terminal esterase L5 (UCHL5), have been indicated to regulate the levels or activities of various oncogene or tumor suppressor proteins through their deubiquitylating activities.^[^ ^21^ ^]^ Development of DUB selective/specific inhibitors is emerging as attractive targets for cancer treatment. Although various DUB inhibitors have been reported in the last decade, relatively little research has examined the anti‐tumor effects of DUB inhibitors in HCC.

O‐GlcNAcylation affects serine and threonine residues of cytoplasmic, nuclear, and mitochondrial proteins, and is a widespread dynamic and reversible post‐translational modification (PTM).^[^ ^22^, ^23^ ^]^ This monosaccharide alteration has been identified as a crucial regulator of numerous significant biological and pathological processes, including signal transduction, protein interactions, and enzymatic activity.^[^ ^24^ ^]^ Unlike most other PTMs, O‐GlcNAcylation is catalyzed by only two conserved enzymes, the O‐GlcNAc transferase (OGT) and O‐GlcNAcase (OGA), for the addition and removal of O‐GlcNAc, respectively.^[^ ^25^ ^]^ It has been referred that aberrant O‐GlcNAc is associated with the malignant properties in cancer cells.^[^ ^26^, ^27^, ^28^ ^]^ A recent study revealed that inhibition of O‐GlcNAcylation results in mitochondria fragmentation and enhances mitophagy, providing an additional source of labile iron and rendering the cell more sensitive to ferroptosis.^[^ ^29^ ^]^

To discover novel therapeutics for HCC, we screened 23 compounds, with the main focus on targeting DUBs. And the compound DUB‐IN‐3, a small molecular inhibitor of USP8, showed the most effective anti‐cancer responses. Further analysis demonstrated that pharmacological inhibition or knockout of USP8 in HCC cells triggered ferroptosis and inhibited tumor growth both in vitro and in vivo. Mechanistically, targeting USP8 destabilized OGT via promoting K48‐specific poly‐ubiquitination process on OGT protein at K117 site, thus decreasing the Ser26 O‐GlcNAcylation of solute carrier family 7, member 11 (SLC7A11) in HCC cells, which is essential for SLC7A11 to import the cystine from the extracellular environment. Targeting USP8 may prove to be a potential target for the treatment of HCC.

## 2 Results

### 2.1 DUB‐IN‐3 Is a Potent Inhibitor of HCC

**Figure 1:**
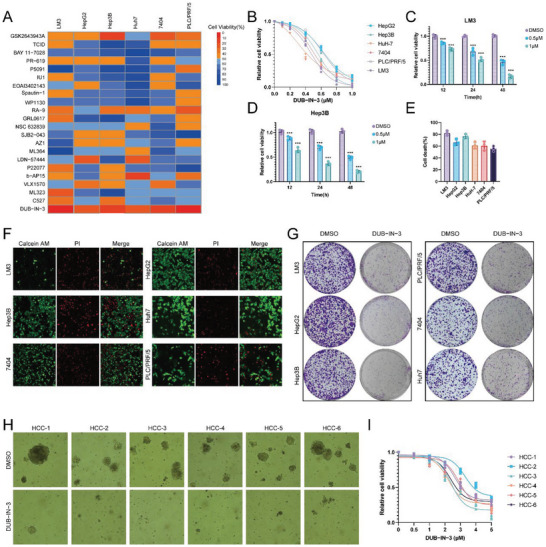
DUB‐IN‐3 is a potent inhibitor of Hepatocellular carcinoma (HCC). A) The heat map displays the growth inhibition rate of six HCC cell lines to the 22 compounds targeting DUBs (1 µm for 48 h, which were selected based on the literature). B) Dose–response curves of six HCC cell lines to DUB‐IN‐3 treatment. All cells were treated with increasing doses of DUB‐IN‐3 for 48 h, and cell viability was determined by CCK8 assay. C,D) Cell viability analysis of DUB‐IN‐3 treatment in two HCC cell lines (LM3 and Hep3B) at the indicated concentrations for the indicated times. E) Cell death analysis of six HCC cell lines treated with DUB‐IN‐3 (1 µm for 48 h). Cell death was determined by propidium iodide staining. F) Calcein/PI staining of HCC cells treated with DUB‐IN‐3 (1 µm for 48 h). G) Crystal violet staining of HCC cells treated with DUB‐IN‐3. HCC cells (5 × 10^4^) were seeded in 6‐well plates, and after 3 days, cells were treated with DMSO or DUB‐IN‐3 (1 µm) for 48 h. Cells were then washed and stained with crystal violet. H) Representative images of PDOs treated with DUB‐IN‐3 (5 µm, 48 h). I). Dose‐response curves of six PDOs to DUB‐IN‐3 treatment. All PDOs were treated with increasing doses of DUB‐IN‐3 for 48 h, and cell viability was determined by CellTiter‐Glo assay. Results shown are representative of 3 independent experiments. Data are represented as mean ± SD of biological triplicates.*p value < 0.05; **p value < 0.01; and ***p value < 0.001.

In order to discover efficient HCC growth inhibitors, we screened 23 compounds that were known to target human DUBs in six HCC cell lines. All six cell lines showed high sensitivity to DUB‐IN‐3, a USP8 inhibitor (**Figure** **1**A,B). We also found that DUB‐IN‐3 decreased the cell viability of LM3 and Hep3B cells in a dose‐ and time‐dependent manner (Figure 1C,D). We next tested the efficacy of DUB‐IN‐3 on cell death via PI staining. As shown in Figure 1E, DUB‐IN‐3 markedly induced the death of HCC cells. Consistently, DUB‐IN‐3 treatment leads to increased cell death as revealed by crystal violet and Calcein AM/PI staining assays (Figure 1F,G). We further established 6 patient‐derived organoids (PDOs) from primary liver cancers and tested the anti‐cancer efficacy of DUB‐IN‐3. We found that DUB‐IN‐3 exhibited excellent cancer‐inhibitory effects in PDO models (Figure 1H,I).

**Figure 2:**
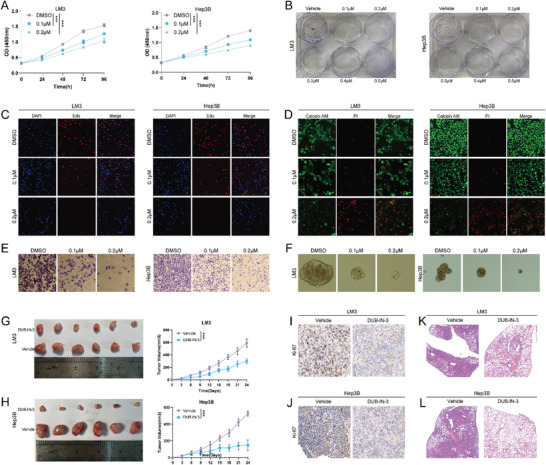
DUB‐IN‐3 inhibits HCC progression both in vitro and in vivo. A) CCK8 assays of cells treated with vehicle or DUB‐IN‐3. B) Colony formation assays of cells treated with vehicle or DUB‐IN‐3. HCC cells were seeded into 6‐well plates (2000 cells per well) and incubated with the indicated concentration of DUB‐IN‐3 for 14 days, cells were washed with PBS and stained with 0.5% crystal violet. C) Edu assays of cells treated with vehicle or DUB‐IN‐3. HCC cells were cultured in 96‐well plates at a density of 1000 cells per well and incubated with the indicated concentration DUB‐IN‐3, after 48 h, Edu assay was performed. D) Calcein/PI staining of cells treated with vehicle or DUB‐IN‐3 (HCC cells were treated with indicated concentration DUB‐IN‐3 for 48 h). E) Cell invasion assay of cells treated with vehicle or DUB‐IN‐3 (HCC cells were treated with indicated concentration DUB‐IN‐3 for 24 h). F) Sphere formation assay of cells treated with vehicle or DUB‐IN‐3. Single cells (2 × 10^3^) were seeded into 6‐well ultra‐low attachment culture plates and incubated with indicated concentration DUB‐IN‐3 for 1 week. Then the spheres were photographed and counted G,H) DUB‐IN‐3 suppressed the growth of HCC xenografts in nude mice. LM3 and Hep3B xenografts treated with vehicle (*n* = 6) or DUB‐IN‐3 (*n* = 6; 5 mg kg^−1^ per day; intraperitoneally). I,J) Representative images of immunohistochemical staining for Ki67. K,L) DUB‐IN‐3 suppressed the lung metastasis of HCC in mice. HCC cells (0.5 × 10^6^) were intravenously injected into each mouse through the tail vein. Mice were treated with vehicle (*n* = 6) or DUB‐IN‐3 (*n* = 6; 5 mg kg^−1^ per day; intraperitoneally). The lungs were harvested 4 weeks after injection. Results shown are representative of three independent experiments. Data are represented as mean ± SD of biological triplicates.*p value < 0.05; **p value < 0.01; and ***p value < 0.001.

To further examine the anti‐cancer efficacy of DUB‐IN‐3, we chose two cell lines, LM3 and Hep3B, which showed higher sensitivity to DUB‐IN‐3 treatment for further analysis. Cell counting kit 8 (CCK8) analysis indicated that DUB‐IN‐3 inhibited the proliferation of HCC cells (**Figure** **2**A). Clone formation assay demonstrated that DUB‐IN‐3 significantly inhibited the clone formation ability of HCC cells (Figure 2B). EdU incorporation assay was further performed to measure the DNA synthesis. Our results indicated that DUB‐IN‐3 inhibited the DNA synthesis of LM3 and Hep3B cells (Figure 2C). Consistently, DUB‐IN‐3 leads to increased cell death as revealed by Calcein AM/PI staining assay (Figure 2D). Transwell assay demonstrated that DUB‐IN‐3 dramatically decreased the invasion capacity of LM3 and Hep3B cells (Figure 2E). We then examined the role of DUB‐IN‐3 in HCC stemness characteristics. It was found that DUB‐IN‐3 significantly reduced the oncosphere formation of LM3 and Hep3B cells (Figure 2F). We next utilized xenograft and tail vein injection lung metastatic models to address the role of DUB‐IN‐3 in HCC in vivo. Our results indicated that DUB‐IN‐3 significantly inhibited tumor growth and metastasis in vivo (Figure 2G–L). As DUB‐IN‐3 is a small molecular inhibitor of USP8, we then investigated the biological functions of USP8 in HCC cells. Consistent with our previous observations of DUB‐IN‐3, knockout of USP8 significantly suppressed the proliferation, invasion, and stemness of HCC cells (Figure S1, Supporting Information). To further determine whether the effects of DUB‐IN‐3 were mediated by USP8, we treated USP8 knocked‐out cells with DUB‐IN‐3. As per our expectations, DUB‐IN‐3 did not suppress the proliferation, invasion, and stemness of USP8‐KO cells (Figure S2, Supporting Information). These data indicated that DUB‐IN‐3 may serve as a novel potent anti‐HCC agent via targeting USP8.

### 2.2 Targeting USP8 Suppresses the Glutathione Metabolism and Confers Ferroptosis of HCC

**Figure 3:**
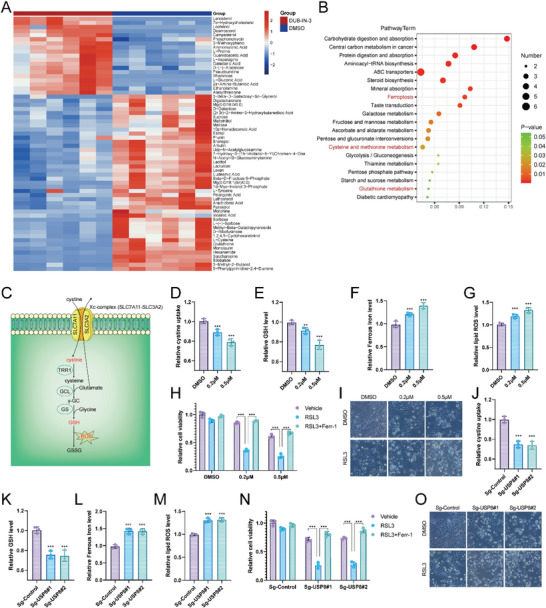
Targeting USP8 suppresses the glutathione metabolism and confers ferroptosis of HCC. A) Heatmap showing significantly differently expressed metabolites between DMSO and DUB‐IN‐3‐treated groups. LM3 cells were treated with DUB‐IN‐3 (0.5 µm), and after 24 h, cells were collected for GC‐MS analysis (*n* = 6). B) The top 20 enriched pathways from integrated pathway analysis of significantly changed metabolites. C) Illustration of the GSH metabolism pathway. D–G) Cystine, GSH, ferrou iron, and lipid ROS levels were quantified in LM3 cells treated with indicated concentration of DUB‐IN‐3 for 24 h. H) LM3 cells were treated with the indicated concentration of DUB‐IN‐3 for 24 h, and then the response of DUB‐IN‐3‐treated LM3 cells to RSL3 (10 µm)±ferrostatin (1 µm) was detected using CCK8 assay. I) Representative images of DUB‐IN‐3‐treated LM3 cells treated with RSL‐3 (10 µm, 24 h). J–M). Cystine, GSH, ferrou iron, and lipid ROS levels were quantified in LM3 cells depleted with USP8. N) CCK8 assay showing the response of USP8‐depletion LM3 cells to RSL3 (10 µm)±ferrostatin (1 µm). O) Representative images of USP8‐depletion LM3 cells treated with RSL‐3 (10 µm, 24 h). Results shown are representative of three independent experiments. Data are represented as mean ± SD of biological triplicates.*p value < 0.05; **p value < 0.01; and ***p value < 0.001.

To explore the underlying mechanisms of the results identified above, we performed gas chromatography with TOF‐mass spectrometry‐based (GC/TOF‐MS‐based) metabolomics analysis. Heatmap analysis demonstrated that DUB‐IN‐3 treatment induced a marked difference in metabolic signatures (**Figure** **3**A). The pathway enrichment of those significantly changed metabolites further demonstrated that many metabolic pathways were influenced in DUB‐IN‐3 treated cells, and we observed that the glutathione (GSH) metabolism pathway, cysteine and methionine metabolism pathway, and ferroptosis pathway were significantly affected (Figure 3B). GSH is reported as one of the most important antioxidants in living organisms, which functions as a critical scavenger of reactive oxygen species (ROS) particularly lipid ROS.^[^ ^30^, ^31^, ^32^ ^]^ GSH metabolism plays an important role in tumor progression, and elevated levels of GSH are associated with increased metastasis.^[^ ^33^ ^]^ Recent studies reported that the cystine/glutamate antiporter system Xc‐/GSH/glutathione peroxidase 4 (GPX4) axis is a critical pathway involved in regulating ferroptosis, a newly defined form of programmed cell death (Figure 3C).

Suppression of system Xc‐, GSH, or cysteine depletion can induce ferroptosis.^[^ ^34^, ^35^, ^36^, ^37^, ^38^ ^]^ Targeting GSH synthesis/utilization is considered to be a potential means of tumor therapeutics. We then concentrated on these metabolic pathways for the mechanism study. As indicated in Figure 3D,E, the critical intermediates of glutathione metabolism, such as cystine and GSH, were significantly decreased in cells treated with DUB‐IN‐3. And ferrous iron level was upregulated upon DUB‐IN‐3 treatment (Figure 3F). As a consequence of GSH exhaustion, these cells accumulated more lipid ROS (Figure 3G). We further assessed the role of DUB‐IN‐3 in ferroptosis by detecting RSL3‐induced ferroptosis with or without ferrostatin‐1 (Ferr‐1), an inhibitor of ferroptosis. It is observed that DUB‐IN‐3 decreased the cell viability under the treatment of RSL3, which could be rescued by Ferrostatin‐1 (Ferr‐1, indicating that the mechanism is specific in cell ferroptosis (Figure 3H,I). Consistently, knockout of USP8 decreased cystine uptake, GSH level, and increased ferrous iron level and lipid ROS (Figure 3J–M). As expected, cells depleted with USP8 were more sensitive to RSL3 treatment (Figure 3N,O). To further investigate whether targeting USP8 induced cell death through ferroptosis, HCC cells treated with DUB‐IN‐3 or sg‐USP8 were incubated with ferrostatin (1 µm) for 24 h, then cell viability was determined using CCK8 assay. We observed that ferrostatin could largely recover the cell death induced by DUB‐IN‐3 or sg‐USP8 treatment, indicating that targeting USP8 at least partly induced cell death through ferroptosis (Figure S3, Supporting Information). Taken together, pharmacological inhibition or knockout of USP8 may suppress glutathione biosynthesis by inhibiting HCC cells absorbing cystine from the extracellular environment and conferring ferroptosis.

### 2.3 USP8 Stabilizes OGT Through the Deubiquitylation Activity

**Figure 4:**
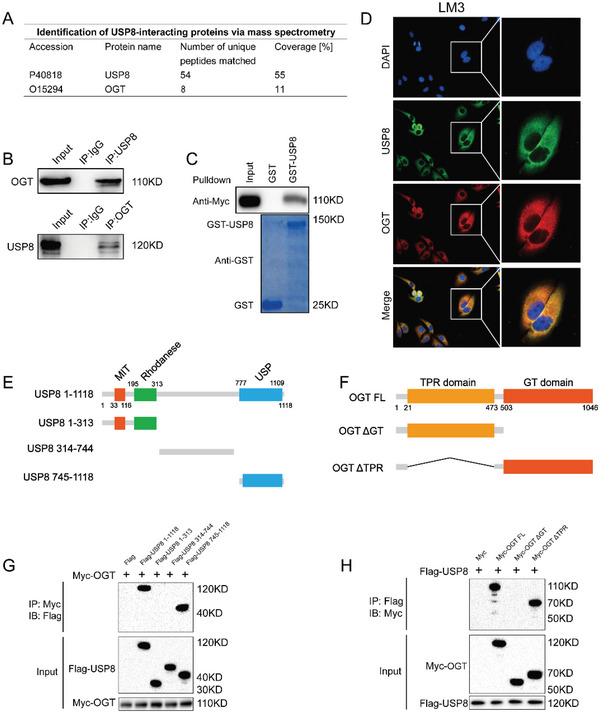
USP8 associates with OGT. A) Mass spectrometry assay of USP8‐associated proteins in LM3 cells was performed, and the specific interactive information between USP8 and OGT was shown. B) Co‐IP assay reveals an association between endogenous USP8 and OGT in LM3 cells. LM3 cells were harvested with RIPA lysis buffer. Co‐IP was performed using antibody as indicated. C) LM3 cells transfected with Myc‐OGT were lysed and the lysates were incubated with GST‐USP8 or GST protein. The interacted OGT was detected by western blot. D) An immunofluorescence assay demonstrated that USP8 and OGT at least partially colocalized in LM3 cells. E,F). USP8 and OGT domain structure and deletion mutants used in the study. J) The USP domain of USP8 interacted with OGT. HEK293 cells were transfected with 2 µg Myc‐OGT together with Flag‐USP8 full‐length or mutants. After 24 h, cells were harvested with NP‐40 lysis buffer. Co‐IP was performed using Myc antibody. The possible interacted USP8 domains were detected by Flag antibody. K) The GT domain of OGT interacted with USP8. HEK293 cells were transfected with 2 µg Flag‐USP8 together with Myc‐OGT full‐length or mutants. After 24 h, cells were harvested with NP‐40 lysis buffer. Co‐IP was performed using Flag antibody. The possible interacted OGT domains were detected by Myc antibody.

To explore the proteins that were potentially regulated by USP8, we then performed immunoprecipitation‐based mass spectrometry (IP‐MS) following ectopic expression of Flag‐USP8 and immunoprecipitation. We noticed that the O‐Linked N‐Acetylglucosamine (GlcNAc) Transferase (OGT) was immunoprecipitated by USP8 (**Figure** **4**A). OGT catalyzes the transfer of a single N‐acetylglucosamine from UDP‐GlcNAc to a serine or threonine residue in cytoplasmic and nuclear proteins resulting in their modification with a beta‐linked N‐acetylglucosamine (O‐GlcNAc).^[^ ^39^ ^]^ Further co‐immunoprecipitation indicated that endogenous USP8 could coimmunoprecipitate with endogenous OGT (Figure 4B). GST‐pull‐down assay showed that USP8 interacted with OGT in vitro (Figure 4C). We then performed an immunofluorescence assay to assess the cellular localization of USP8 and OGT. The results of immunostaining demonstrated that USP8 and OGT colocalized both in the cytosol of HCC cells (Figure 4D). Additionally, deletion analysis demonstrated that the GT domain of OGT physically interacted with the USP domain of USP8 (Figure 4E–H).

**Figure 5:**
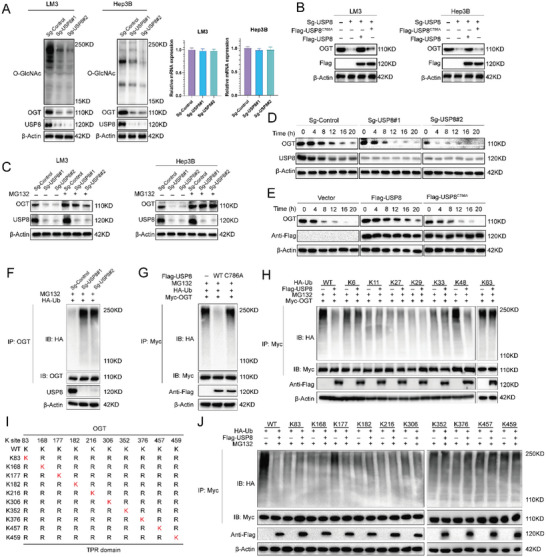
USP8 stabilizes de‐polyubiquitylates OGT. A) USP8 depletion decreased OGT protein level without affecting mRNA expression of OGT. B) USP8 WT or C786A was introduced into HCC depleted with USP8 using sgRNAs. OGT levels were measured. C) HCC cells depleted with USP8 were treated with or without the proteasome inhibitor MG132 (10 µm, 6 h), and then proteins were analyzed. D) LM3 cells depleted with USP8 were treated with cycloheximide (10 µg mL^−1^), and collected at the indicated times for western blot. E) Half‐life analysis of OGT in HEK293 cells transfected with the indicated plasmids. F) LM3 cells depleted with USP8 were treated with MG132 for 6 h before collection. OGT was immunoprecipitated with anti‐OGT and immunoblotted with anti‐HA. G) Immunoblotting was used to detect the ubiquitination of OGT in HEK293T cells co‐transfected with Myc‐ OGT, HA‐Ubiquitin, and Flag‐USP8 (wild‐type or C786A). H) HA‐WT, K6, K11, K27, K29, K33, K48 or K63 Ub were co‐transfected with Myc‐OGT and Flag‐USP8 into HEK293T cells. After treatment with 10 µm MG132 for 6 h, cell lysates were subjected to ubiquitination assay, and the ubiquitination level of OGT detected by HA antibody. I) A schematic diagram of OGT and its mutants. J) Immunoblotting to detect the ubiquitination of OGT mutants in HEK293T cells co‐transfected with Myc‐OGT mutants, USP8, and HA‐Ub.

Since USP8 is a member of the ubiquitin‐specific processing protease family, we hypothesized that USP8 may regulate the turnover of OGT through the Ub‐proteasome pathway. We found that depletion of USP8 markedly decreased OGT levels without influence on its mRNA abundance (**Figure** **5**A), and the decrease could be reversed by the addition of proteasome inhibitor MG132 or overexpression wild‐type (WT) USP8, but not its catalytically inactive mutant (USP8‐C786A) (Figure 5B,C). Consistently, DUB‐IN‐3 decreased OGT protein levels in a dose‐dependent manner. While in cells depleted with USP8, DUB‐IN‐3 treatment could not influence the expression of OGT (Figure S4A,B, Supporting Information). To prove that USP8 affects OGT stability, LM3 cells were treated with the protein synthesis inhibitor cycloheximide (CHX). The half‐life of OGT was shortened in cells depleted of USP8 (Figure 5D). And OGT stability was increased upon overexpression USP8‐WT but not USP8‐C786A (Figure 5E). These results indicated that USP8 stabilizes OGT in cells through the Ub‐proteasome pathway.

We further examined whether OGT is a substrate of USP8. As illustrated in Figure 5F, the depletion of USP8 significantly increased the level of ubiquitinated OGT. Inhibition of USP8 by DUB‐IN‐3 enhanced OGT ubiquitination in a dose‐dependent manner (Figure S4C, Supporting Information). Conversely, ectopic expression of USP8‐WT, but not USP8‐C786A, markedly decreased OGT ubiquitylation in cells (Figure 5G). In vitro ubiquitylation assay indicated that USP8 directly decreased OGT ubiquitylation (Figure S4D, Supporting Information). In vivo ubiquitylation assays showed that USP8 removed the ubiquitin chain of OGT in a time‐ and dose‐dependent manner (Figure S4E, Supporting Information). We also performed a ubiquitination assay with a series of mutant ubiquitin (K6, K11, K27, K29, K33, K48, and K63) to investigate which type of ubiquitin chain of OGT was deubiquitylated by USP8. The result indicated that USP8 could efficiently remove the K48‐linked ubiquitin chain from the OGT protein (Figure 5H).

To further determine the region of OGT that is modulated by USP8, we performed a ubiquitination assay using the ΔGT, ΔTPR, or full‐length constructs of OGT and USP8. It is found that USP8 mainly cleaved the polyubiquitin chains on the TPR domain of OGT, but exerted no effect on the GT domain of OGT (Figure S4F, Supporting Information). Based on the analysis of three bioinformatic tools (UbiSite, BDM‐PUB, and UbPred), a total of 10 lysines were predicted as potential ubiquitination sites in the TRP domain of OGT protein. To determine the specific sites of OGT protein that are deubiquitinated by USP8, we mutated the lysine residues of OGT (Figure 5I). A ubiquitination assay indicated that K117 was the key site on OGT deubiquitinated by USP8 (Figure 5J). We then analyzed the correlation between USP8 and OGT in HCC samples. IHC analysis indicated that USP8 and OGT were both upregulated in HCC samples (Figure S5A, Supporting Information). Besides, we observed a positive correlation between USP8 and OGT protein levels in human HCC samples (Figure S5B, Supporting Information). Survival analysis revealed that high expression of USP8 was related to poor prognosis (Figure S5C, Supporting Information). Taken together, these results indicated that USP8 was a regulator of OGT and was a prognostic marker.

### 2.4 Phosphorylation of USP8 is Required for the Interaction with OGT

**Figure 6:**
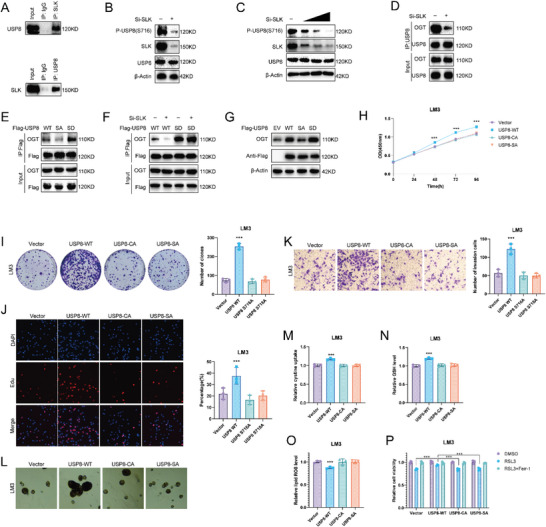
SLK phosphorylates USP8. A) Co‐IP assay reveals an association between endogenous USP8 and SLK in LM3 cells. LM3 cells were harvested with RIPA lysis buffer. Co‐IP was performed using antibody as indicated. B) The expression of USP8 serine 716 phosphorylation was detected in LM3 cells. C) The expression of USP8 serine 716 phosphorylation was detected in LM3 cells transfected with increasing amounts of SLK siRNA. D) Co‐IP assay with anti‐USP8 antibody in LM3 cells treated as indicated. E) The Co‐IP analysis of OGT and Flag‐USP8 WT, S716A, or S716D in HEK293T cells. F) LM3 cells transfected with Flag‐USP8 WT or S716D mutants were treated with siControl or siSLK. G) LM3 cells were transfected with Flag‐USP8 WT, S716A, or S716D mutants, and OGT protein levels were detected. H) CCK8 assays of LM3 cells transfected with USP8 WT, S716A or C786A. I) Colony formation assays of LM3 cells transfected with USP8 WT, S716A, or C786A. J) Edu assays of LM3 cells transfected with USP8 WT, S716A, or C786A. K) Cell invasion assay of LM3 cells transfected with USP8 WT, S716A, or C786A. L) Sphere formation assay of LM3 cells transfected with USP8 WT, S716A, or C786A. M–O). Cystine, GSH, and lipid ROS levels were quantified in LM3 cells transfected with USP8 WT, S716A or C786A. P) CCK8 assay showing the response of LM3 cells to RSL3 (10 µm)±ferrostatin (1 µm). Results shown are representative of three independent experiments. Data are represented as mean ± SD of biological triplicates.*p value < 0.05; **p value < 0.01; and ***p value < 0.001.

Recent studies reported that DUBs could be regulated by several post‐translational modifications in tumors, which can influence the deubiquitinase activity and stability of the DUBs.^[^ ^40^, ^41^, ^42^ ^]^ Our mass spectrometry (MS) analysis revealed that USP8 was phosphorylated at Ser716 residues (Figure S6A, Supporting Information). Interestingly, we discovered that STE20 Like Kinase (SLK) was co‐purified with Flag‐USP8 (Figure S5B, Supporting Information). Endogenous Co‐IP experiments were conducted in LM3 cell lines and verified the interaction between SLK and USP8 (**Figure** **6**A). Depletion of SLK by siRNA significantly reduced the S716 phosphorylation of USP8 without influence on its protein levels (Figure 6B,C).

We next sought to determine whether SLK‐mediated USP8 phosphorylation influenced its association with OGT. As expected, the association between USP8 and OGT was markedly decreased upon SLK depletion (Figure 6D). Consistently, the phosphorylation‐null USP8 (S716A) decreased while the phosphomimetic USP8 (S716D) mutant increased its association with OGT (Figure 6E). We then depleted SLK in LM3 cells with ectopic expression of the wild‐type (WT) USP8 or USP8‐S716D, and it was found that SLK depletion decreased the ability of USP8‐WT to bind to OGT (Figure 6F). We also observed that wild‐type and phosphomimetic USP8, but not the phosphorylation‐null USP8, increased OGT protein levels (Figure 6G).

To investigate the role of USP8 phosphorylation in HCC cells, we stably expressed USP8 WT and S716A in HCC cells, the empty vector and catalytically inactive mutant C786A were used as negative control. Consistent with the results identified above, USP8 WT promoted the proliferation, invasion, and stemness of HCC cells, but the C786A and S716A mutants lost these abilities (Figure 6H–L; Figure S7A–D, Supporting Information). USP8 WT, but not C786A and S716A mutants, enhanced cystine uptake and GSH levels of HCC cells, while decreasing lipid ROS levels and cell sensitivity to RSL3 treatment (Figure 6M–P; Figure S7E–H, Supporting Information).

We also observed that Protein Phosphatase 1 Catalytic Subunit Alpha (PPP1CA) was co‐purified with Flag‐USP8 from our mass spectrometry analysis (Figure S6B, Supporting Information). Endogenous Co‐IP experiments revealed the interaction between PPP1CA and USP8 (Figure S6C, Supporting Information). Further analysis indicated that PPP1CA mediated the dephosphorylation of USP8 (Figure S6D, Supporting Information). As expected, ectopic expression of PPP1CA dephosphorylated USP8 and decreased the interaction between USP8 and OGT (Figure S6E,F, Supporting Information). These results suggest that phosphorylation of USP8 is required for the interaction with OGT.

### 2.5 OGT O‐GlcNAcylated SLC7A11 at Ser26 in HCC Cells

**Figure 7:**
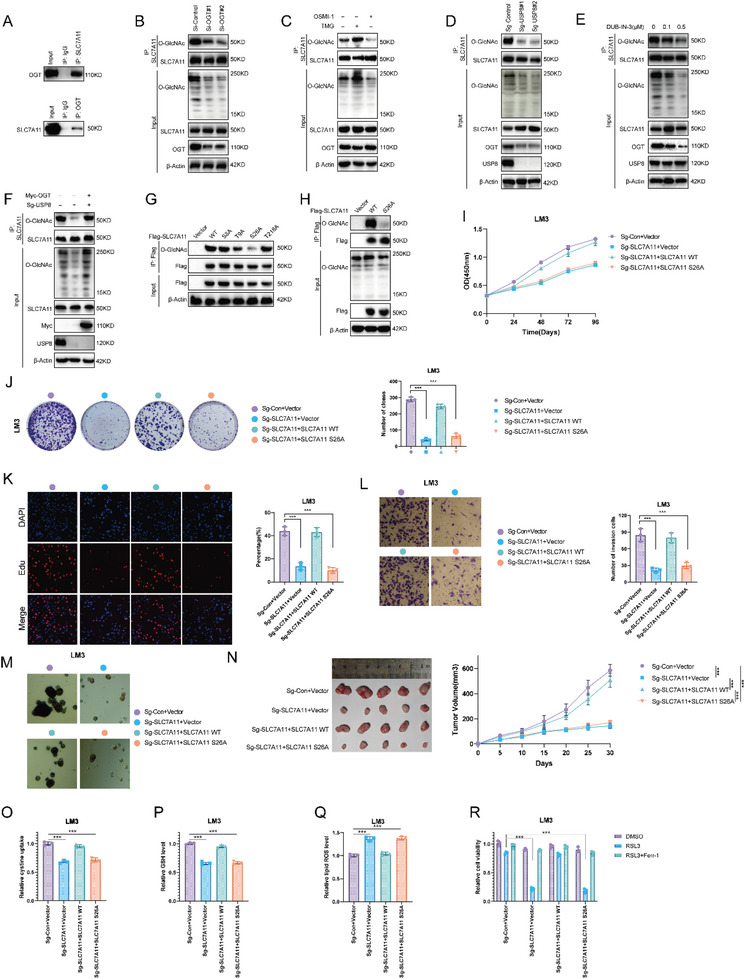
OGT O‐GlcNAcylated SLC7A11 at Ser26 in HCC cells. A) Co‐IP assay reveals an association between endogenous OGT and SLC7A11 in LM3 cells. LM3 cells were harvested with RIPA lysis buffer. Co‐IP was performed using antibody as indicated. B) Depletion of OGT decreased the O‐GlcNAcylation of SLC7A11. SLC7A11 was immunoprecipitated with anti‐SLC7A11 and O‐GlcNAcylated SLC7A11 was immunoblotted with anti‐O‐GlcNAcylation. C) Western blot showing TMG (10 µm, 24 h) and OSMI‐1 (50 µm, 24 h) treatment regulates SLC7A11 O‐GlcNAcylation level. D) Depletion of USP8 decreased the O‐GlcNAcylation of SLC7A11. E) DUB‐IN‐3 treatment decreased the O‐GlcNAcylation of SLC7A11. F) USP8 regulated the O‐GlcNAcylation of SLC7A11 through OGT. G,H) SLC7A11 was O‐GlcNAcylated at serine 26 in cells. 293T cells were transfected with the indicated plasmids, and SLC7A11 O‐GlcNAcylation was analyzed by immunoprecipitation with anti‐FLAG antibody and western blot with the indicated antibodies. I) CCK8 assays of LM3 cells. J) Colony formation assays of LM3 cells K) Edu assays of LM3 cells. L) Cell invasion assay of LM3 cells. M) Sphere formation assay of LM3 cells. N) In vivo xenografts generated from LM3 cells transfected with the indicated plasmids. HCC cells (1 × 10^6^) were injected into the right dorsal flank of each mouse. Tumor sizes were measured every 5 days until the end of the experiment. O–Q) Cystine, GSH, and lipid ROS levels were quantified in LM3 cells. R) CCK8 assay showing the response of LM3 cells to RSL3 (10 µM)±ferrostatin (1 µm). Results shown are representative of three independent experiments. Data are represented as mean ± SD of biological triplicates.*p value < 0.05; **p value < 0.01; and ***p value < 0.001.

Our results indicated that USP8 deubiquitinated OGT and affected the cystine uptake of HCC cells. Since cystine is imported by the cystine/glutamate antiporter system xc– from the extracellular environment, we set out to determine whether SLC7A11, a specific and functional subunit of system xc–, was regulated by the USP8‐OGT axis. Co‐immunoprecipitation assay indicated the association between OGT and SLC7A11 under physiological conditions (**Figure** **7**A). Immunoprecipitated SLC7A11 was then detected using anti‐O‐GlcNAc antibody. It was found that immunoprecipitated SLC7A11 was O‐GlcNAcylated, and depletion of OGT decreased the O‐GlcNAcylation of SLC7A11 (Figure 7B). Higher global O‐GlcNAcylation increased SLC7A11 O‐GlcNAcylation in TMG (OGA inhibitor) treated cells, while cells inhibited by OSMI‐1 (OGT inhibitor) showed the opposite phenomenon (Figure 7C). Consistent with our expectations, O‐GlcNAcylation of SLC7A11 decreased in response to treatment with the DUB‐IN‐3 or USP8 sg‐RNAs (Figure 7D,E). In addition, overexpression of OGT could rescue the O‐GlcNAcylation of SLC7A11 in USP8‐KO cells (Figure 7F). These results indicated that USP8 regulated the O‐GlcNAcylation of SLC7A11 through OGT.

To identify the O‐GlcNAcylation site on SLC7A11, we first used an online bioinformatic tool (https://services.healthtech.dtu.dk/) to predict the potential O‐GlcNAcylation sites. As a result, a total of 4 residues were identified (S8, T9, S26, and T218). To determine which residue(s) is the major glycosylation site in SLC7A11, we mutated each of the four residues to alanine. We found that mutation of S26, but not the other three residues, reduced the O‐GlcNAcylation signal to a large degree, suggesting that S26 is the major glycosylation site (Figure 7G,H).

To further understand the effect of O‐GlcNAcylation on SLC7A11 activity, we knockout SLC7A11 (SLC7A11‐KO) in HCC cells. After that, SLC7A11‐WT and SLC7A11‐S26A were separately transfected into SLC7A11‐KO cells. As expected, SLC7A11 knockout suppressed the proliferation, invasion, and stemness of HCC cells, overexpression of SLC7A11‐WT recovered the effects induced by SLC7A11 knockout, but SLC7A11‐S26A lost the ability (Figure 7I–N; Figure S8A–D, Supporting Information). Consistently, SLC7A11‐WT, but not SLC7A11‐S26A, enhanced cystine uptake and GSH levels of HCC cells, while decreased lipid ROS levels and cell sensitivity to RSL3 treatment (Figure 7O–R; Figure S8E–H, Supporting Information). Taken together, these results indicated that SLC7A11 is O‐GlcNAcylated by OGT in HCC cells and O‐GlcNAcylation of SLC7A11 is essential for its cystine‐uptake activity.

To further confirm that USP8 regulates cystine uptake and intracellular levels of glutathione through the OGT‐SLC7A11 axis, we ectopically expressed OGT or SLC7A11 in USP8 knockout LM3 cells. We observed that the cystine uptake ability was mostly reversed by overexpression of OGT or SLC7A11 in USP8 knockout LM3 cells. While increased OGT or SLC7A11 expression only partially recovered the intracellular levels of glutathione and lipid ROS levels (Figure S9A–C, Supporting Information). Consistently, cell death induced by RSL3 was only partially recovered by ectopic expression of OGT and SLC7A11, indicating some other proteins regulated by USP8 and OGT may also contribute to ferroptosis resistance (Figure S9D, Supporting Information).

## 3 Discussion

HCC is a common and extremely aggressive malignancy, it is usually undetectable and how to treat patients with advanced stages of HCC is still a therapeutic challenge. The effects of conventional treatments are limited in the treatment of patients with HCC, while recent improvements in understanding the genetic and molecular mechanisms of HCC hold promise for targeted therapy for this disease. Growing evidence has indeed shown that DUBs are involved in the development and progression of cancer. Various small molecule DUB inhibitors are considered promising anticancer agents for the development of novel cancer therapies. However, the potential roles of DUB inhibitors in the treatment of HCC are currently unknown.

USP8 is a member of the USP protease family. USP8 expression is frequently upregulated in various types of cancer and related to poor prognosis,^[^ ^43^ ^]^ including melanoma, gastric cancer, cholangiocarcinoma, breast cancer, and lung cancer.^[^ ^44^, ^45^, ^46^, ^47^, ^48^ ^]^ High expression of USP8 is usually associated with the high proliferation and metastasis abilities of tumors.^[^ ^49^ ^]^ It is reported that USP8 decreases the efficacy of anti‐PD‐L1/PD‐1 immunotherapy via reshaping an inflamed tumor microenvironment (TME). In several murine tumor models, anti‐PD‐L1/PD‐1 immunotherapy combination with USP8 inhibitor significantly increases the infiltration of CD8+ T cells and suppresses tumor growth. The mechanistic study revealed that inhibition of USP8 increases PD‐L1 protein expression by promoting Lys63‐linked polyubiquitin chains of PD‐L1 to antagonize Lys48‐linked ubiquitination in a manner dependent on TRAF6, thus inhibiting the degradation of PD‐L1. In addition, USP8 inhibition triggers MHC‐I expression and innate immune response through activating the NF‐κB signaling.^[^ ^50^ ^]^ In breast cancer, USP8 directly deubiquitinates and stabilizes the type II TGF‐β receptor TβRII. USP8 also promotes tumor metastasis, invasion, and epithelial‐mesenchymal transition in response to TGF‐β/SMAD signaling. USP8 increases TβRII+ circulating extracellular vesicles, thus inducing T cell exhaustion and chemoimmunotherapy resistance.^[^ ^51^ ^]^ USP8 inhibitor combination with PD‐1/PD‐L1 immunotherapy may be a promising approach to enhance anti‐tumor efficacy.

In the present study, we observed that pharmacological inhibition or knocked‐out of USP8 significantly suppressed the progression of HCC. We first examined the anticancer efficacy of DUB‐IN‐3 in HCC cells. Our results demonstrated that inhibition of USP8 reduced the proliferation and clone formation, and induced ferroptosis of HCC. In vivo using xenograft and tail vein injection mouse models indicated that inhibition of USP8 significantly reduced tumor growth and inhibited lung metastasis. To explore the underlying mechanism, we performed TOF‐mass spectrometry‐based metabolomics analysis. Intriguingly, DUB‐IN‐3 treatment caused significant changes in metabolic pathways. We further observed that the glutathione metabolism pathway, cysteine and methionine metabolism pathway, and ferroptosis pathway were significantly affected. GSH plays a main role in ferroptosis and maintains the balance of intracellular redox.^[^ ^52^ ^]^ ROS was physiologically produced by aerobic cells, an excess formation of ROS leads to cell damage and death. In order to prevent irreversible damage, cells develop adaptive responses to restore redox homeostasis, such as the upregulation of antioxidant defense systems.^[^ ^53^ ^]^ GSH level is increased in various types of tumors, elevated GSH level is essential for cell cycle progression and is associated with cellular proliferation and metastatic activity.^[^ ^54^, ^55^, ^56^ ^]^ In this study, the GSH level was markedly decreased in cells treated with DUB‐IN‐3 or USP8 depletion. While ROS was accumulated in these cells.

Cysteine serves as the rate‐limiting precursor for the biosynthesis of GSH. Most cancer cells mainly rely on obtaining cysteine from the extracellular environment through nutrient transporters.^[^ ^57^ ^]^ We also quantified cystine uptake levels in HCC cells. As indicated, both DUB‐IN‐3‐treated and USP8‐KO cells exhibited much lower levels of cystine uptake, suggesting USP8 is important for cells to sustain the activity of absorbing cystine from the extracellular environment. System xc^−^ functions as a cystine/glutamate antiporter, mediating cystine entry into the cell in exchange for glutamate.^[^ ^58^ ^]^ Some ferroptosis‐inducing drugs, such as sorafenib and erastin, could inhibit SLC7A11‐mediated cysteine transport into cells to promote ferroptosis.^[^ ^59^ ^]^

The cystine‐glutamate transporter system xc^−^ is a heterodimer consisting of the light chain subunit SLC7A11 and the heavy chain subunit SLC3A2. The extracellular cystine is imported into the cell through SLC7A11, whereas SLC3A2 anchors SLC7A11 to the plasma membrane and maintains SLC7A11 protein stability.^[^ ^60^ ^]^ In addition, we observed that SLC7A11 was O‐GlcNAcylated by the USP8‐OGT axis in HCC cells, and O‐GlcNAcylation of SLC7A11 is essential for its functions to absorb cystine. Mechanistically, USP8 may act as a potent DUB responsible for OGT deubiquitination and stabilization, which in turn O‐GlcNAcylates SLC7A11 in HCC. First, USP8 and OGT interacted with each other. Co‐IP analysis identified the association between OGT and USP8. Second, USP8 decreased OGT polyubiquitination and promoted its protein stabilization in a DUB activity‐dependent manner. USP8 deletion markedly decreased OGT protein levels, and this effect could be reversed by the addition of the proteasome inhibitor MG132 or overexpression of USP8‐WT, but not its catalytically inactive mutant USP8^C786A^. Upon inhibition of protein synthesis by cycloheximide, USP8 depletion significantly decreased the half‐life time of OGT protein. Ectopic expression of USP8‐WT, but not USP8C^786A^, markedly decreased OGT ubiquitylation, indicating that the catalytical activity is essential for USP8 to regulate OGT protein levels. Consistently, the C786A mutant of USP8 could not promote the proliferation, invasion, and stemness of HCC cells (Figure 6H–L). Only USP8 WT, but not the C786A mutant, enhanced cystine uptake and GSH levels of HCC cells, while decreasing lipid ROS levels and cell sensitivity to RSL3 treatment. Further analysis indicated that K117 is the key site on OGT deubiquitinated by USP8. We observed that USP8 significantly decreased K48‐linked polyubiquitination from OGT. As polyubiquitination through K48 of Ub generally results in proteasomal degradation,^[^ ^58^, ^59^ ^]^ USP8 may maintain the stability of OGT by removing the K48‐linked ubiquitin chain from the OGT protein. Third, we found that the phosphorylation of USP8 at S716 was required for the interaction with OGT. The phosphorylation‐null USP8 lost its ability to interact with OGT and could not promote the progression of HCC. Fourth, OGT was responsible for the O‐GlcNAcylation of SLC7A11 at S26 site. O‐GlcNAc is an abundant, dynamic, and inducible post‐translational modification occurring on serine and threonine residues, which can modulate various protein functions.^[^ ^61^, ^62^ ^]^ Growing evidence indicates that O‐GlcNAcylation is altered in cancer. Increased total O‐GlcNAcylation is a general characteristic of cancer cells, that regulates multiple cancer cell phenotypes.^[^ ^63^, ^64^, ^65^, ^66^ ^]^ O‐GlcNAcylation has been reported to play multiple roles in ferroptosis.^[^ ^67^ ^]^ O‐GlcNAcylation of c‐Jun stimulates GSH synthesis and reduces ROS accumulation, thus inhibiting ferroptosis.^[^ ^68^ ^]^ O‐GlcNAcylation antagonizes Ser127 phosphorylation to inhibit the degradation of YAP, resulting in elevated Fe2+ concentration and lipid peroxidation.^[^ ^69^ ^]^ O‐GlcNAcylation of ZEB1 also promotes the transcriptional activity of adipogenesis‐related genes FASN and FADS2, leading to increased synthesis of PUFAs and activating ferroptosis.^[^ ^63^ ^]^ O‐GlcNAcylation of the ferritin heavy chain at S179 inhibits its interaction with NCOA4, the ferritinophagy receptor, thereby preventing cells from ferroptosis.^[^ ^29^ ^]^ In the present study, we found that ectopic expression of SLC7A11‐S26A mutant could not recover the cystine uptake in SLC7A11‐KO cells. Indicating O‐GlcNAcylation of SLC7A11 is necessary for its activity. A recent study demonstrated that the suppression of USP8 sensitizes cancer cells to ferroptosis through up‐regulating ferritinophagy and intracellular iron levels in a SQSTM1‐NCOA4‐ferritin dependent manner.^[^ ^70^ ^]^ We also observed that ectopic expressed OGT or SLC7A11 in USP8 knockout LM3 cells only partially recovered the cell death induced by RSL3, indicating some other proteins regulated by USP8 and OGT may also contribute to ferroptosis resistance.

A previous study reported that inhibition of the O‐GlcNAcylation of the ferritin heavy chain at S179 promotes its interaction with NCOA4, the ferritinophagy receptor, thereby accumulating labile iron for ferroptosis. The O‐GlcNAcylation of ZEB1 at Ser555 site enhances its stabilization and nuclear translocation and induces lipogenesis‐associated gene transcription activity, which ultimately results in lipid peroxidation‐dependent mesenchymal pancreatic cancer cell ferroptosis.^[^ ^63^ ^]^ However, the O‐GlcNAcylation of SLC7A11 has not been investigated yet. Our present study reveals a previously unrecognized post‐translational modification of SLC7A11 in HCC, which was mainly regulated by USP8‐OGT axis. USP8 was responsible for OGT de‐ubiquitination and stabilization, which O‐GlcNAcylated SLC7A11 to maintain its functions.

**Figure 8:**
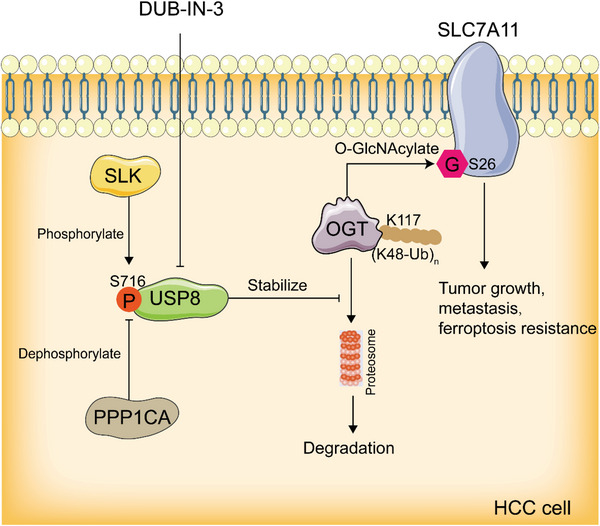
Graphic summary of USP8/OGT/SLC7A11 pathway. USP8 deubiquitinates and stabilizes OGT by removing the K48‐linked ubiquitin chain at K117 site, OGT O‐GlcNAcylates SLC7A11 at S26 site. Phosphorylation of USP8 at S716 is required for the interaction with OGT.

In conclusion, we demonstrated that targeting USP8 decreased the stability of OGT, inhibited the progression, and induced ferroptosis of HCC. Mechanically, USP8 deubiquitinated and stabilized the O‐linked N‐Acetylglucosamine Transferase OGT by removing the K48‐linked ubiquitin chain, which then O‐GlcNAcylated SLC7A11 at S26 site. O‐GlcNAcylation of SLC7A11 was necessary for its activity uptake cystine from the extracellular environment. Most importantly, phosphorylation of USP8 at S716 was required for the interaction with OGT (**Figure** **8**). Our findings provide new insights into the roles of USP8 in regulating glutathione metabolism and ferroptosis in HCC and suggest that targeting USP8 is a promising therapeutic strategy for HCC.

## 4 Experimental Section

### Cell Culture

The human liver cancer cell lines LM3, HepG2, Huh‐7, 7404, Hep3B, PLC/PRF/5, and human embryonic kidney HEK293T cells were obtained from the Procell Life Science&Technology Co., Ltd. (Wuhan, China). All cell lines were authenticated by the cell banks with short tandem repeat analysis. All cell lines were cultured in Dulbecco's modified Eagle's medium (DMEM, 41965, Life Technologies) supplemented with 10% fetal bovine serum (FBS, Gibco, Life Technologies, 10270). All cells were cultured at 37 °C, in 5% CO2 humid atmosphere.

### Plasmids and Generation of USP8 Knockout Cell Lines

The SLC7A11, OGT, and USP8 and mutant plasmids were obtained from Hanbio Biotechnology Co., Ltd. (Shanghai, China). The HA‐K6, ‐K11, ‐K27, ‐K29, ‐K33, ‐K48, ‐K63, and ‐Ub plasmids were acquired from Addgene. USP8 knockout cells were generated using the LentiCRISPRv2 system. The single‐guide RNA (sgRNA) sequences (sg‐USP8#1: TCGAAGAATGCAGGATTATC and sg‐USP8#2 CACATTGGCTAAAGGCTCTT) used in the experiment were synthesized by Genepharma (Shanghai, China). Annealed sgRNA oligo was ligated into the lentiCRISPRv2 vector obtained from Hanbio Biotechnology Co., Ltd. (Shanghai, China). The constructed lentiCRISPR vector containing USP8 sgRNA was transduced into HEK293 cells with the packing plasmids psPAX2 and pMD2 using Lipofectamine 2000. The virus‐containing medium was collected 48 and 72 h after transfection, and cell debris was removed by passing through a 0.45 µm filter. LM3 and Hep3B cells were infected with the lentivirus for 24 h and then selected with 1 µg mL^−1^ puromycin.

### Co‐Immunoprecipitation Assay

Cells were washed with pre‐chilled phosphate‐buffered saline (PBS) and lysed with RIPA extraction reagent (Meilun, China) supplemented with protease inhibitors (Meilun, China). Cell lysates were pre‐cleared and incubated with the indicated antibody overnight at 4 °C, the antibody associated with the protein complex was then incubated with protein A/G PLUS‐Agarose beads for an additional 2 h. The beads were washed with PBS three times and boiled at 100  °C for 10 min to reverse crosslinking before SDS‐PAGE immunoblotting analysis.

### Protein Stability Assay

To measure the half‐life of OGT, cells were treated with 100 µm protein synthesis inhibitor cycloheximide (Sigma–Aldrich) for indicated times. Western blot was performed to measure protein levels.

### In Vivo Deubiquitination Assay

For in vivo deubiquitination assay, HA‐Ub, Myc‐OGT, Flag‐USP8, or Flag‐USP8 ^C786A^ plasmid were transfected into HEK293T cells for 48 h. Cells were then treated with 10 µm MG132 (MCE) for 6 h. Then, cells were washed with pre‐chilled phosphate‐buffered saline (PBS) and lysed with RIPA extraction reagent. HA‐ubiquitinated OGT was isolated using an anti‐Myc antibody. The ubiquitination level of OGT was detected by Western blotting with an anti‐HA antibody. In HCC cells, HA‐Ub plasmid was co‐transfected into USP8‐KO LM3 cells. HA‐ubiquitinated OGT was isolated using an anti‐OGT antibody. The ubiquitination level of OGT was detected by Western blotting with an anti‐HA antibody.

### In Vitro Deubiquitination Assay

HEK293T cells were transfected with empty vector or Flag‐OGT and HA‐Ub plasmids. Forty‐eight hours after transfection, ubiquitinated OGT proteins were immunoprecipitated with anti‐FLAG M2 affinity gels (Sigma–Aldrich). The ubiquitinated YAP proteins were incubated with bacterially purified GST or GST‐ USP8 proteins in deubiquitination buffer (50 mm NaCl, 50 mm Tris‐HCl, 10 mm dithiothreitol, 1 mm EDTA, 5% glycerol, pH 8.0) at 37 °C for 2 h. After the reaction, the reaction mix was supplemented with SDS to a final concentration of 2%, boiled at 95 °C to dissociate all protein‐protein interactions, diluted 10–20 times via the deubiquitination buffer, and then immunoprecipitated by Flag beads before the beads were boiled again and assessed for the HA‐ubiquitin level by WB.

### Western Blot Analysis

NP‐40 lysis buffer supplemented with protease inhibitors (Meilun, China) was used to extract total protein. BCA Reagent (Thermo Scientific, Rockford, IL, USA) was used to measure the protein concentration. An SDS‐polyacrylamide gel was used to separate the total proteins and transferred proteins to a 0.45 µm PVDF membrane (Millipore, USA). The primary antibodies used for western blot analysis are listed as follows: SLC7A11 (Proteintech, 26864‐1‐AP), OGT (Proteintech, 11576‐2‐AP), USP8 (Proteintech, 67321‐1‐Ig), O‐GlcNAcylation (Santa Cruz, sc‐59623) GAPDH (Proteintech, 60004‐1‐Ig), Flag (Proteintech, 66008‐4‐Ig), Myc (Proteintech, 60003‐2‐Ig), HA (Proteintech, 51064‐2‐AP) antibodies. For the antibody against p‐USP8(S716), the rabbit polyclonal anti‐phosphorylated Ser716 of USP8 antibodies were generated by immunizing rabbits with phosphopeptide. In brief, New Zealand white rabbits were immunized with the synthetic phosphopeptide QIPAERDREPSKLKR(pS)YSSPDITQAIQEEE (corresponding to residues 701–720 of human USP8) conjugated to keyhole limpet haemocyanin. The rabbits were immunized on day 1, 3, 28, and 42 and euthanized 7 days after the final immunization. The sera were collected and affinity purified with the phosphopeptide. Signals were detected and visualized using ECL (Meilun, China) and ChemiDocMP imager (Bio‐Rad).

### Analysis of Lipid ROS

For the measurement of lipid ROS, cells were washed with PBS and incubated with PBS containing 10 µm C11‐BODIPY (581/591) (Thermo Fisher Scientific, #D3861) at 37 °C for 30 min in a cell culture incubator. The cells were then washed with PBS to remove excess C11‐BODIPY and cells were harvested for flow cytometer analysis (Fortessa, BD Biosciences) with fluorescein isothiocyanate (FITC) green channel and Texas red channel.

### GSH Assay

The Glutathione Assay Kit (Beyotime Biotechnology) was used for the detection of total GSH according to the manufacturer's instructions. Briefly, cultured HCC cells were washed with PBS and collected in centrifuge tubes, and then 3 volumes of protein removal reagent S solution were added immediately. After being fully vortexed, the samples were frozen and thawed twice in liquid nitrogen and 37 °C water. The mixture was left to stand for 5 min at 4 °C. Then the samples were centrifugated at 4 °C, 10 000 g for 10 min, and the supernatant was collected for the measurement of total GSH.

### In Vivo Tumorigenesis Assay

Animal experiments were conducted according to the protocols approved by an ethnic committee of Xiangya Hospital. BALB/c nude mice aged 4 weeks were obtained from Beijing HFK Bioscience Co., Ltd. (Beijing, China). HCC cells (1 × 10^6^) were resuspended in 100 µL DMEM and injected subcutaneously into the flanks of BALB/c nude mice aged 6 weeks. A vernier caliper was used to measure the tumor sizes and the same was recorded every other day until the end of the experiment.

### Live/Dead Cell Staining

The treated cells were washed with PBS and then incubated with 2 µm Calcein‐AM (Meiun, China) and 2 µm PI solution (Meiun, China) at 37 °C for 15 min, and photographed under a fluorescence microscope (N2‐DMi8, Leica).

### Cell Viability Assay

Cell viability was measured with CCK8 assay. HCC cells (5 × 10^3^) were seeded into duplicate wells of 96‐well microplates, drugs were added after cell adhesion, and cell viability was assessed by CCK8 assay at indicated time points. Cell viability was calculated by normalizing the absorbance at 450 nm of the experimental groups to that of the negative control group.

### Sphere Formation Assay

Single cells (2 × 10^3^) were seeded into 6‐well ultra‐low attachment culture plates (Corning, USA) in serum‐free DMEM/F12 supplemented with B27 (1:50), 20 ng mL^−1^ EGF, and 20 ng mL^−1^ bFGF. Two weeks later, the spheres were photographed and counted.

### Mass Spectrometry Analysis

OE Biotech Co., Ltd. was responsible for IP‒MS and the detection process has been described previously.^[^ ^71^ ^]^ For metabolomics analysis, LM3 cells were plated and treated with DUB‐IN‐3 (1 µm) or DMSO for 12 h. Cells were collected by scraping, and cell pellets from these two sets of samples were immediately flash‐frozen in liquid nitrogen and stored at −80 °C until extraction. The changes in metabolites were examined by GC/TOF‐MS‐based metabolomics as previously described.^[^ ^72^ ^]^ All data analysis was performed by OE Biotech Co., Ltd. (Shanghai, China).

### Statistical Analysis

Statistical analysis was performed using Prism 9.5 (GraphPad, USA) with a two‐tailed student's *t*‐test or ANOVA test. Multiple comparison was carried out using Bonferroni's adjustment. The results were repeated in at least three independent experiments and are shown as the mean‐standard deviation. * indicates *p* < 0.05, ** indicates *p* < 0.01, *** indicates *p* < 0.001, and **** indicates *p* < 0.0001.

## Conflict of Interest

The authors declare no conflict of interest.

## Author Contributions

L.Z. and Y.T. designed and supervised the research. J.T., G.L., K.H., L.Z., D.X., S.L., L.X., and Y.T. performed research and provided helpful discussions. J.T., G.L., L.X., L.Z., D.X., S.L., and Y.T. analyzed and interpreted the data. D.X. conducted pathology evaluations. All authors reviewed and edited the manuscript. Y.T. contributed to all aspects of the study. G.L. performed animal model experiments. J.T. had a primary role in interpreting and organizing the data as well as writing the manuscript.

## Supporting information

Supporting Information

## Data Availability

The data that support the findings of this study are available from the corresponding author upon reasonable request.
