# Supplementary material for: Targeting USP8 Inhibits O‐GlcNAcylation of SLC7A11 to Promote Ferroptosis of Hepatocellular Carcinoma via Stabilization of OGT
Source: Adv Sci (Weinh). 2023 Oct 22;10(33):2302953. doi: 10.1002/advs.202302953 (PMC10667802; doi:10.1002/advs.202302953)
Supplement: Supplementary file 1 — Supporting Information [file ADVS-10-2302953-s001.pdf]

ADVANCED SCIENCE
Open Access

Supporting Information

for *Adv. Sci.*, DOI 10.1002/advs.202302953

Targeting USP8 Inhibits O-GlcNAcylation of SLC7A11 to Promote Ferroptosis of Hepatocellular Carcinoma via Stabilization of OGT

Jianing Tang, Guo Long, Kuan Hu, Desheng Xiao, Shuang Liu, Liang Xiao, Ledu Zhou* and Yongguang Tao**

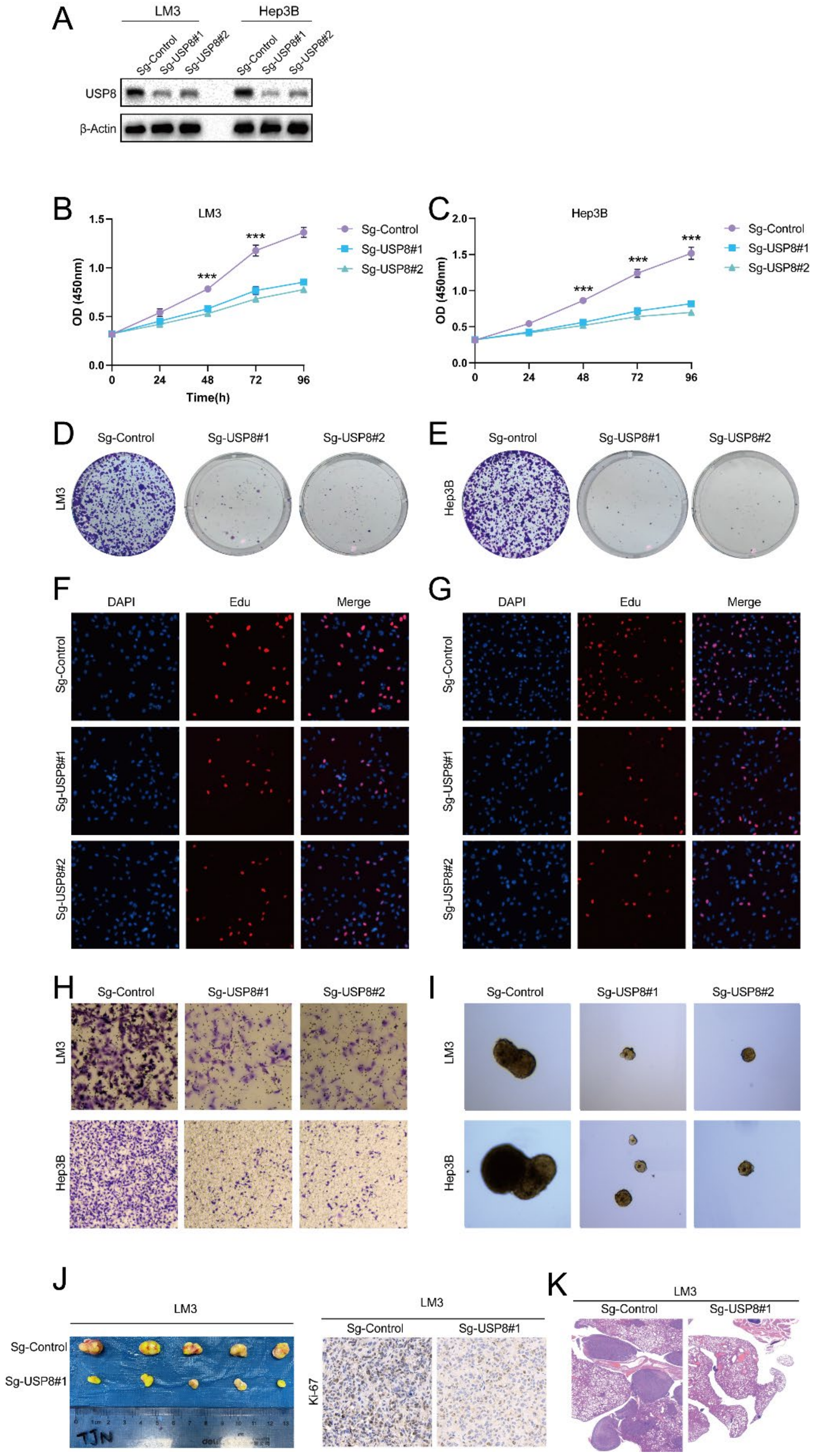

Figure S1. USP8 knockout suppresses the progression of HCC cells. (A). knockout efficiency of USP8 (B, C). CCK8 assays of LM3 and Hep3B cells. (D, E) Colony formation assays of LM3 and Hep3B cells. (F, G) Edu assays of LM3 and Hep3B cells. (H). Cell invasion assay of cLM3 and Hep3B cells. (I). Sphere formation assay

of LM3 and Hep3B cells. (J) Depletion of USP8 suppressed the growth of HCC xenografts in nude mice. 1×10^6 LM3 cells were injected to the right dorsal flank of each mouse. Tumor sizes were measured every 5 days until the end of the experiment. (J). Depletion of USP8 suppressed the lung metastasis of HCC in mice. 0.5×10^6 HCC cells were intravenously injected into each mouse through the tail vein. The lungs were harvested 4 weeks after injection.

Results shown are representative of 3 independent experiments. Data are represented as mean ± SD of biological triplicates.*, *P value < 0.05; **, P value < 0.01; ***, P value < 0.001.*

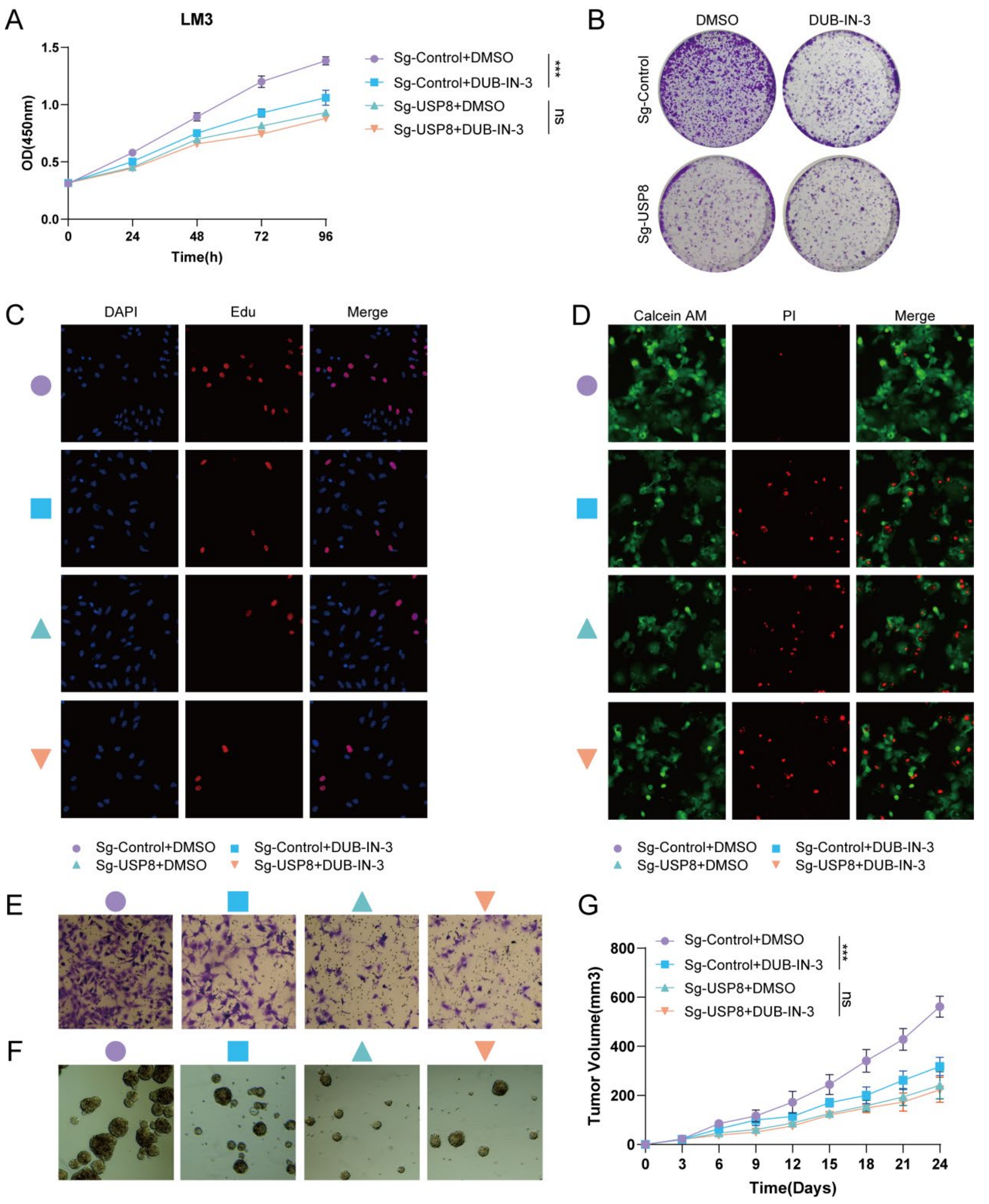

Figure S2. DUB-IN-3 inhibits HCC progression via USP8. (A) CCK8 assays of wild type and USP8-KO LM3 cells treated with vehicle or DUB-IN-3. (B) Colony formation assays of wild type and USP8-KO LM3 cells treated with vehicle or DUB-IN-3. (C) Edu assays of wild type and USP8-KO LM3 cells treated with vehicle or DUB-IN-3. (D). Calcein/PI staining of wild type and USP8-KO LM3 cells treated with vehicle or DUB-IN-3. (E). Cell invasion assay of wild type and USP8-KO LM3 cells treated with vehicle or DUB-IN-3. (E). Sphere formation assay of wild type and USP8-KO LM3 cells treated with vehicle or DUB-IN-3. (G, H) DUB-IN-3 suppressed

the growth of wild type LM3 xenograft in nude mice. LM3 xenograft treated with vehicle (n = 6) or DUB-IN-3 (n = 6; 5 mg/kg/day; intraperitoneally).

Results shown are representative of 3 independent experiments. Data are represented as mean ± SD of biological triplicates.*, *P value < 0.05; **, P value < 0.01; ***, P value < 0.001.*

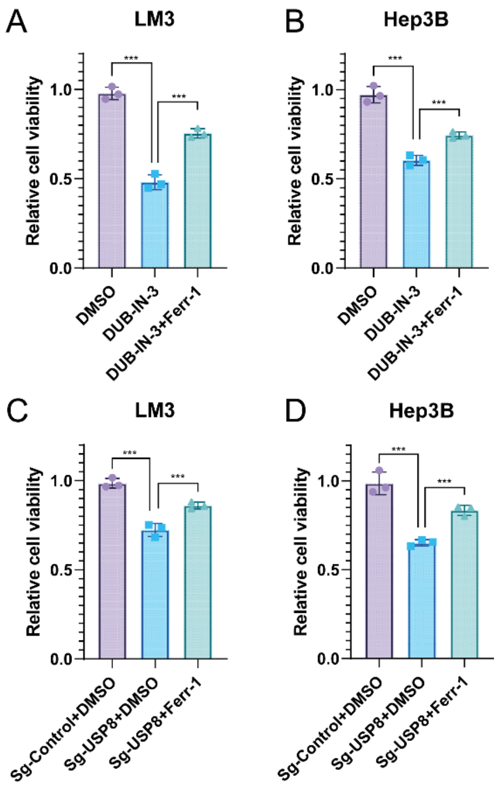

Figure S3. Targeting USP8 induces ferroptosis. (A. B). DUB-IN-3 induced cell death could be largely recovered by ferrostatin. HCC cells were treated with 0.5 μM DUB-IN-3±ferrostatin (1 μM) for 48 h. (C. D). USP8 depletion induced cell death could be largely recovered by ferrostatin. HCC cells depleted with USP8 were treated with ferrostatin (1 μM) for 48 h.

Results shown are representative of 3 independent experiments. Data are represented as mean ± SD of biological triplicates.*, *P value < 0.05; **, *P value < 0.01; ***, *P value < 0.001*.

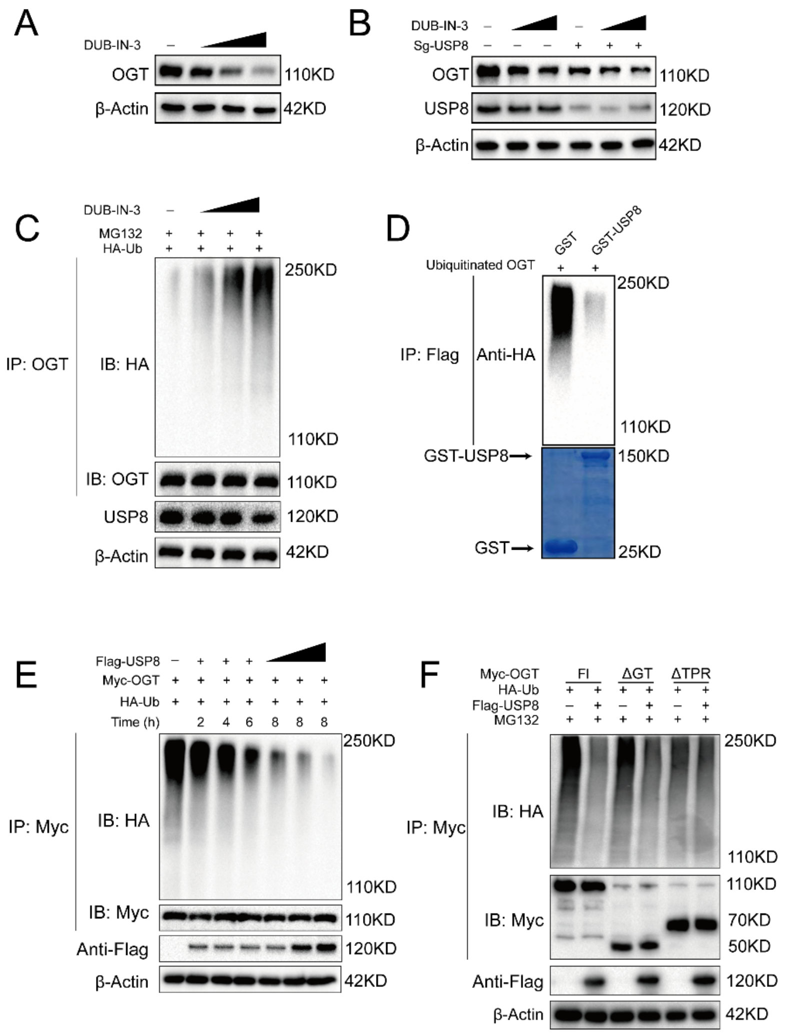

Figure S4. USP8 de-polyubiquitylates OGT. (A). DUB-IN-3 decreased OGT protein levels in a dose-dependent manner. LM3 cells were treated with 0, 0.1, 0.2, 0.5 μM DUB-IN-3 for 24h. (B). DUB-IN-3 decreased OGT protein levels via USP8. LM3 cells depleted with USP8 were treated with DUB-IN-3, and then proteins were analyzed. (C). USP8 de-ubiquitinated OGT *in vitro.* Ubiquitinated OGT was purified from HEK293T cells transfected with HA-Ub, and Flag- OGT plasmids using anti-Flag affinity purification method. The ubiquitinated OGT was incubated with GST-tagged USP8 protein in 20 μl reaction buffer containing at 37 °C for 2 h. The reactions were terminated by boiling in 1×SDS sample buffer and analyzed using IB with anti-HA antibodies. (D). DUB-IN-3 promoted the ubiquitin chain of OGT in a time-dependent manner. LM3 cells were treated with 0, 0.1, 0.2, 0.5 μM DUB-IN-3 for 24h. (E). USP8 removed the ubiquitin chain of OGT in a time- and dose-dependent manner. (F). Immunoblotting to detect the ubiquitination of the OGT

deletion mutants (FL, ΔGT and ΔTPR) in HEK293T cells co-transfected with Myc-OGT (FL, ΔGT and ΔTPR), HA-Ub and USP8.

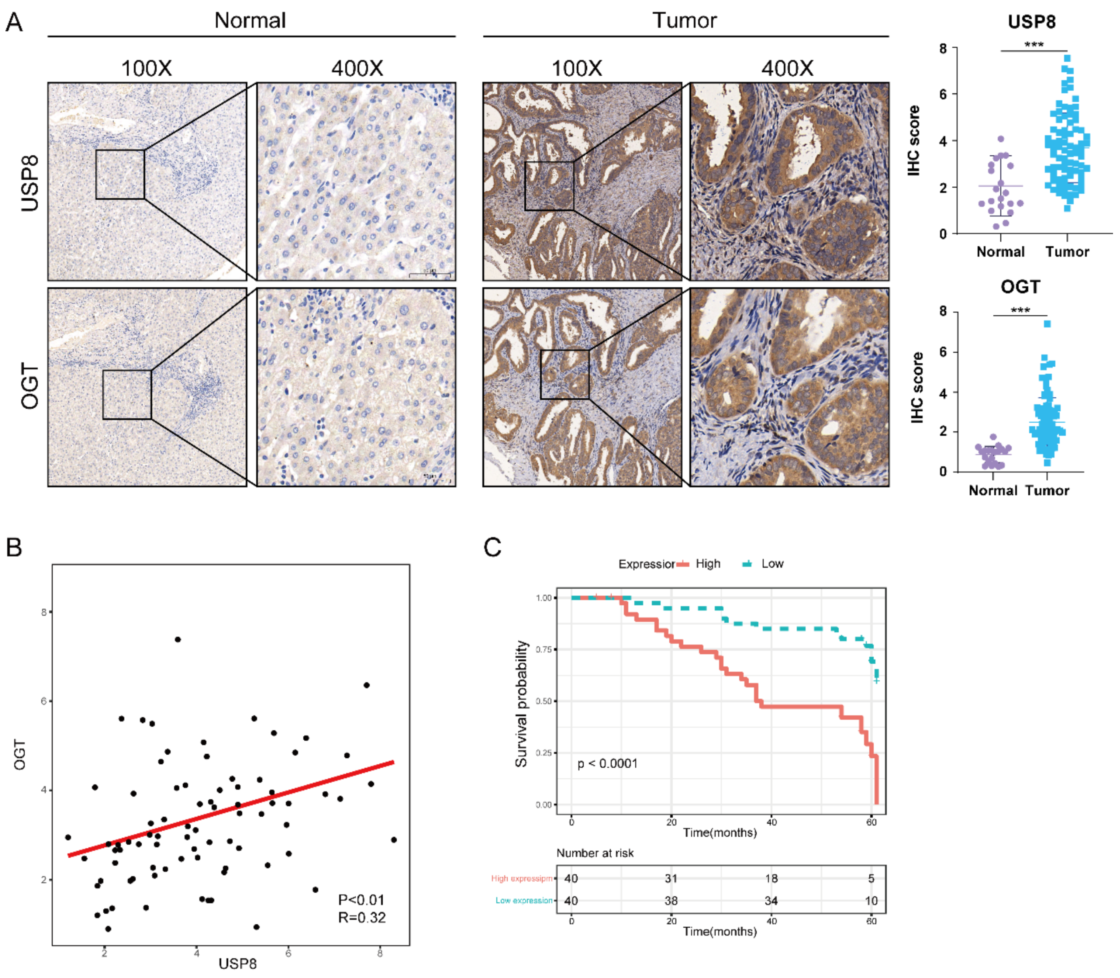

Figure S5. USP8 correlates with OGT protein levels in human HCC samples. (A). USP8 and OGT was upregulated in HCC. 100 samples were used for IHC analysis, including normal adjacent cancerous tissues (n=20) and HCC tissues (n=80). Specific primary antibodies against OGT (Proteintech, 11576-2-AP), USP8 (Proteintech, 67321-1-Ig) were used for IHC. (B). USP8 correlated OGT in human HCC samples. (C). High expression of USP8 was associated with poor prognosis.

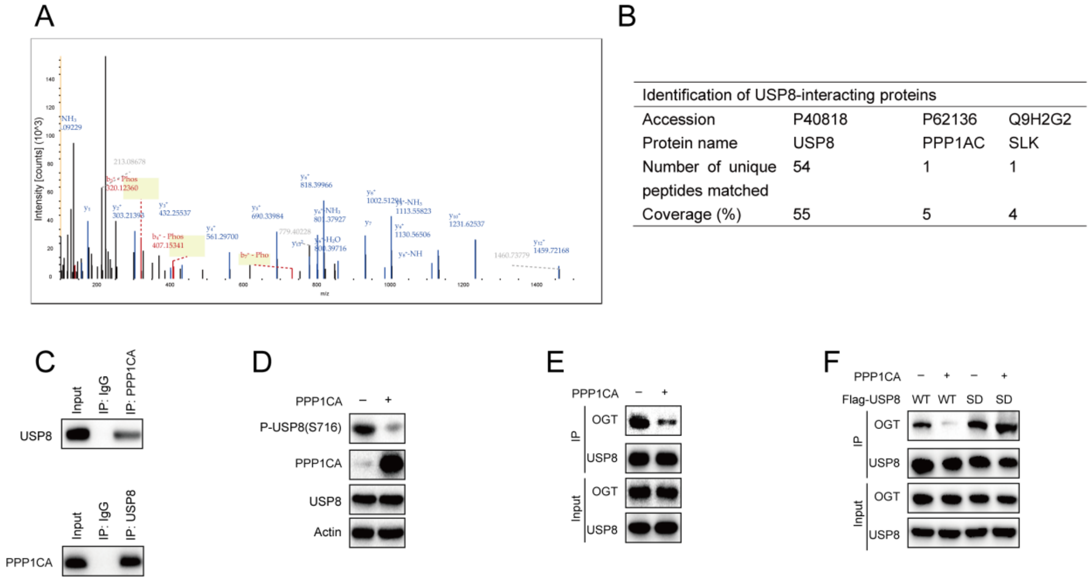

Figure S6. USP8 is phosphorylated in HCC cells. (A). Mass spectrometry analysis revealed lysine residue S716 of USP8 was phosphorylated in LM3 cells. (B). Mass spectrometry assay of USP8-associated proteins in LM3 cells was performed, and the specific interactive information between USP8, SLK and PPP1CA was shown. (C). Co-IP assay reveals association between endogenous USP8 and PPP1CA in LM3 cells. LM3 cells were harvested with RIPA lysis buffer. Co-IP was performed using antibody as indicated. (D). The expression of USP8 serine 716 phosphorylation was detected in LM3 cells transfected with PPP1CA. (E). Co-IP assay with anti-USP8 antibody in LM3 cells treated as indicated. (F). LM3 cells transfected with Flag-USP8 WT or S716D mutants were transfected with empty vector or PPP1CA.

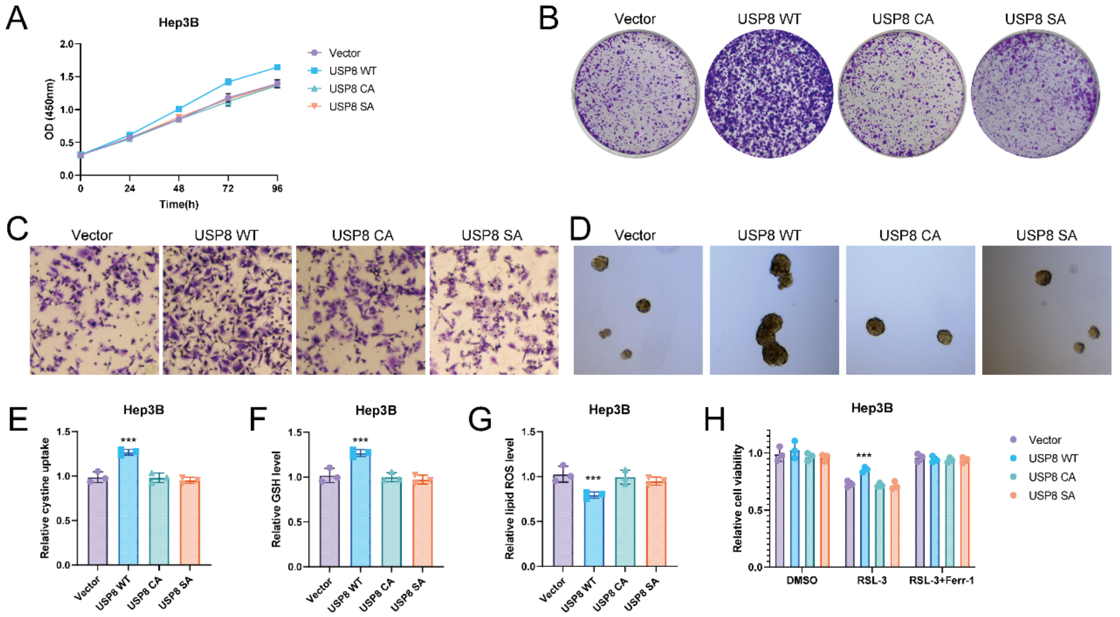

Figure S7. USP8 promotes tumor progression and confers ferroptosis resistance. (A) CCK8 assays of Hep3B cells transfected with USP8 WT, S716A or C786A. (B) Colony formation assays of Hep3B cells transfected with USP8 WT, S716A or C786A. (C). Cell invasion assay of Hep3B cells transfected with USP8 WT, S716A or C786A. (D). Sphere formation assay of Hep3B cells transfected with USP8 WT, S716A or C786A. (E-G). Cystine (E), GSH (F), and lipid ROS levels (G) were quantified in Hep3B cells transfected with USP8 WT, S716A or C786A. (H) CCK8 assay showing the response of Hep3B cells to RSL3 (10 μM)±ferrostatin (1 μM).

Results shown are representative of 3 independent experiments. Data are represented as mean ± SD of biological triplicates.*, *P value < 0.05; **, P value < 0.01; ***, P value < 0.001.*

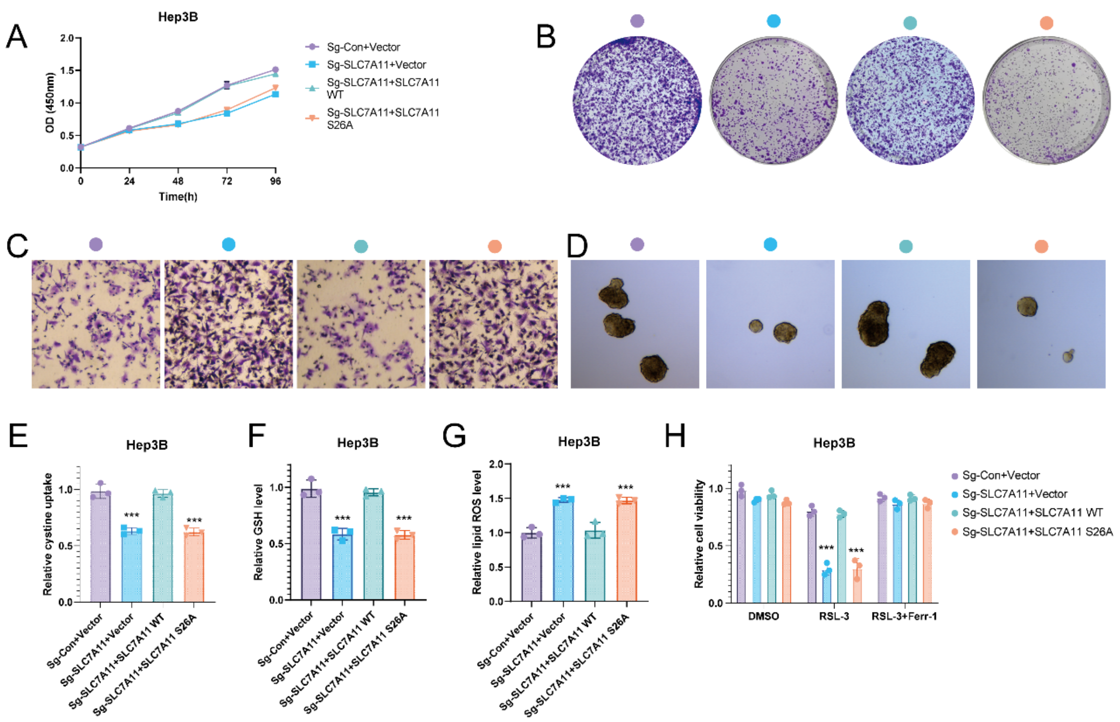

Figure S8. SLC7A11 promotes tumor progression and confers ferroptosis resistance. (A) CCK8 assays of Hep3B cells. (B) Colony formation assays of Hep3B cells. (C). Cell invasion assay of Hep3B cells. (D). Sphere formation assay of Hep3B cells. (E-G). Cystine (E), GSH (F), and lipid ROS levels (G) were quantified in Hep3B cells. (H) CCK8 assay showing the response of Hep3B cells to RSL3 (10 μM) ±ferrostatin (1 μM).

Results shown are representative of 3 independent experiments. Data are represented as mean ± SD of biological triplicates.*, *P value < 0.05; **, *P value < 0.01;* ***, *P value < 0.001.*

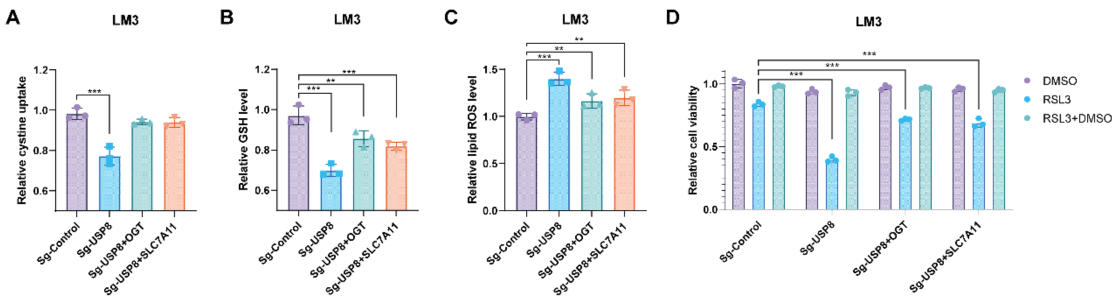

Figure S9. USP8 confers ferroptosis resistance through OGT-SLC7A11 axis. Cystine (A), GSH (B), and lipid ROS levels (C) were quantified in LM3 cells. (D) CCK8 assay showing the response of LM3 cells to RSL3 (10 μM)±ferrostatin (1 μM).

Results shown are representative of 3 independent experiments. Data are represented as mean ± SD of biological triplicates.*, *P value < 0.05; **, P value < 0.01; ***, P value < 0.001.*
